# Pulmonary Alveolar Proteinosis: A Very Rare Disease and the Role of the Cardiac Surgeon in its Treatment 

**Published:** 2015-04-03

**Authors:** Zargham Hossein Ahmadi, Hamid Ghaderi, Seyedeh Adeleh Mirjafari, Tahereh Parsa

**Affiliations:** 1 *Chronic Respiratory Diseases Research Center, National Research Institute of Tuberculosis and Lung Diseases (NRITLD), Shahid Beheshti University of Medical Sciences, Tehran, Iran. 1956944413. Tel: +98 21 27122590-1. Fax: + 98 21 22037591. Email: ahmadiz@sbmu.ac.ir.*; 2 *Department of Cardiovascular Surgery, Shahid Modarress Hospital, Shahid Beheshti University of Medical sciences, Saadat Abad, Tehran, Iran. 1998734383. Tel: +98 21 2208 3106. Fax: +98 21 22074101. E-mail: hghaderi@razi.tums.ac.ir.*; 3 *Brain and Spinal Injury Research Center (BASIR), Imam Khomeini Hospital Complex, End of Keshavarz Blv, Tehran, Iran. 1419733141. Tel: +98 21 66581561. Fax: +98 21 66938885. E-mail: adeleh_60@yahoo.com.*; 4 *Telemedicine Research Center, National Research Institute of Tuberculosis and Lung Diseases (NRITLD), Shahid Beheshti University of Medical Sciences, Tehran, Iran. 1956944413. Tel: +98 21 27122590-1. Fax: + 98 21 22037591.Email: tparsa849@yahoo.com.*

A 5-year-old boy who had progressive dyspnea of 6 months' duration was found to have pulmonary alveolar proteinosis (PAP) by lung biopsy. 

PAP is a rare diffuse intra-alveolar lung disease first described by Rosen et al.^1^ in 1958 and characterized by an accumulation of lipoproteineceous material in the alveoli.^2^ Bronchoalveolar lavage is considered the best treatment for PAP patients. However, our patient's small airways precluded double-lumen intubation and necessitated lung lavage under extracorporeal life support.

Accordingly, through the cannulation of the right carotid artery and the right internal jugular vein, extracorporeal membrane oxygenation was established (Figure 1). Over a period of 18 hours, lung lavage was performed 28 times and gradually the fluid leaving the bronchial tree became clear (Figures 2 and 3).

After the procedure, the patient was transferred to the Intensive Care Unit, where he was extubated at 8 hours postoperatively. The postoperative course was uneventful, and the patient was discharged on the 4^th^ postoperative day in a satisfactory condition.

**Figure 1:**
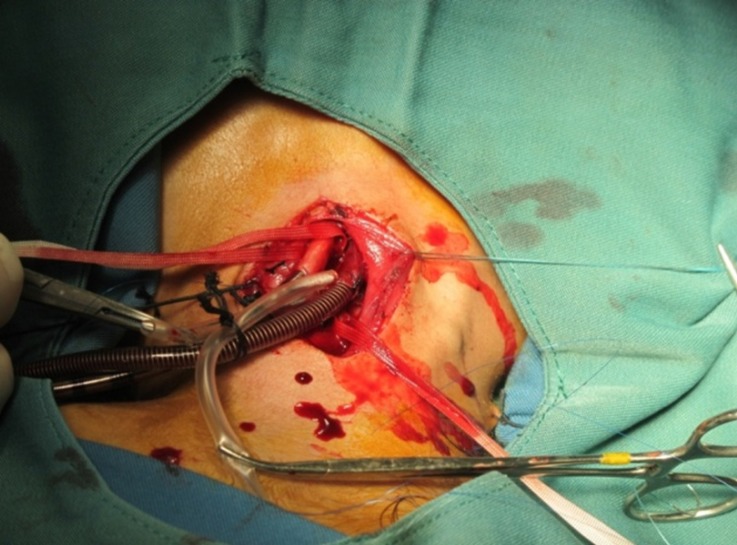
Cannulation of the right carotid artery and the right internal jugular vein for extracorporeal membrane oxygenation

**Figure 2:**
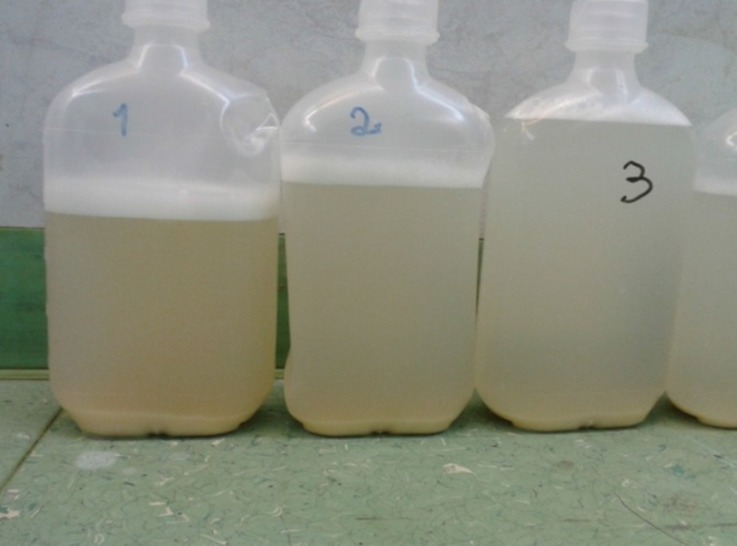
Milky appearance of the first bronchoalveolar lavage

**Figure 3:**
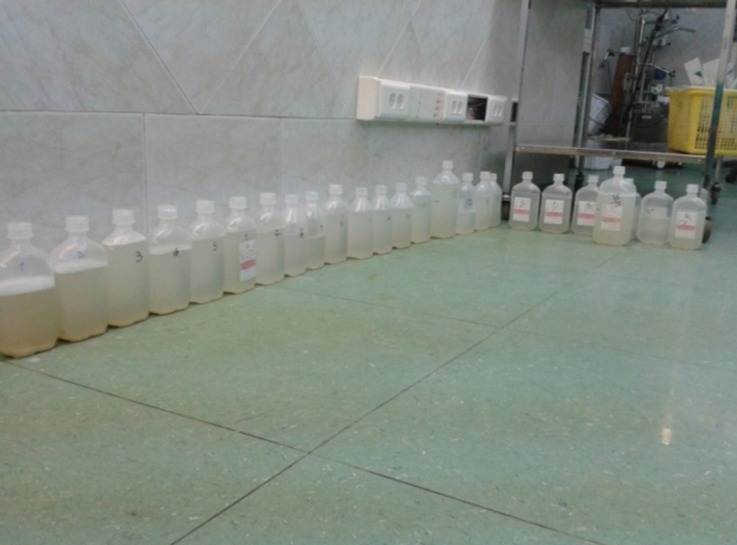
Total amount of fluid extracted from the patient via bronchoalveolar lavage

## References

[1] Akin MR, Nguyen GK (2004). Pulmonary alveolar proteinosis. Pathol Res Pract.

[2] Moulton SL, Krous HF, Merritt TA, Odell RM, Gangitano E, Cornish JD (1992). Congenital pulmonary alveolar proteinosis: failure of treatment with extracorporeal life support. J Pediatr.

